# Paraoxonase-1 gene Q192R and L55M polymorphisms and risk of cardiovascular disease in Egyptian patients with type 2 diabetes mellitus

**DOI:** 10.1186/s40200-014-0125-y

**Published:** 2014-12-20

**Authors:** Dalia El-Lebedy, Mona Kafoury, Dalia Abd-El Haleem, Alshaymaa Ibrahim, Eman Awadallah, Ingy Ashmawy

**Affiliations:** Department of Clinical and Chemical Pathology, Medical Research Division, National Research Center, Al-Bohouth Street, Cairo, 12311 Egypt

**Keywords:** Paraoxonase-1 gene, Cardiovascular disease, Type 2 diabetes mellitus

## Abstract

**Background:**

Increased oxidative stress or an impaired antioxidant defense mechanism may play a crucial role in the onset and progression of atherosclerosis. Recently, Paraoxonase −1 (PON1) which accounts for most of the antioxidant effect of high density lipoprotein (HDL) cholesterol has been presented as a potential therapeutic agent against atherosclerosis development. Allele frequencies for PON1 gene that influence enzyme concentration as well as activity differ greatly among ethnic groups and data from several studies showed ethnic variations in the interpretation of cardiovascular disease (CVD) associated with PON1 polymorphisms. In this work, we investigated PON1 Q192R and L55M polymorphisms in Egyptian patients with type 2 diabetes mellitus (T2DM) and its association with CVD.

**Methods:**

The study included 184 subjects classified into 3 groups; T2DM, T2DM + CVD, and healthy controls. PON1 polymorphisms were genotyped by real-time PCR and PON1 concentration was assayed in serum by ELISA (enzyme linked immunesorbent assay).

**Results:**

Genotype and allele frequencies of Q192R were significantly different between controls and diabetic patients. Frequency of QQ genotype was significantly higher in healthy controls, while QR and RR genotypes were significantly higher in diabetic patients (p = 0.02). Frequency of 55LL and LM genotypes were significantly higher in patients than in controls (p = 0.009). Q192R polymorphism associated with CVD in our diabetic patients (p = 0.01) and with low serum PON1 concentration (p = 0.04). Multiple logistic regression analysis revealed significant correlations between 192R and other independent CVD risk factors.

**Conclusion:**

PON1 192R and 55 L alleles are associated with T2DM. Q192R polymorphism is associated with CVD and lower serum enzyme concentration and might represents a novel risk factor for CVD in Egyptian patients with T2DM.

## Introduction

Diabetes mellitus (DM) is a chronic life-threatening disease and is the second leading cause of death worldwide with at least 1 in 10 deaths among adults between 35 and 64 years old is attributable to diabetes [1]. Cardiovascular disease (CVD) is the most common cause of morbidity and mortality among diabetic patients. People with diabetes are 2 to 4 times more likely to develop coronary artery disease (CAD), 5 times more likely to develop peripheral vascular disease (PVD), and 2 to 6 times more likely to develop myocardial infarction (MI) or stroke than people without diabetes [2]. Several factors such as dyslipidemia, obesity, smoking, exercise, alcohol intake, oxidative stress and genetic variants have been identified as risk factors for CVD in type 2 diabetes (T2DM) [3].

Recently, “dysfunctional HDL” or “proinflammatory HDL” was indicated as a proatherogenic factor [4]. The protective function of HDL against atherogenesis could be partly explained by its main constituent, PON1 (paraoxonase-1) [5]. PON1 is a 355 amino acid glycoprotein which is synthesized in the liver and secreted into the blood where it associates with HDL and accounts for most of its antioxidant effect to prevent oxidation of LDL [6,7].

Increased oxidative stress or an impaired antioxidant defense mechanism may play a crucial role in the onset and progression of atherosclerosis [8]. Since reduced PON1 concentration and/or activity might have a deleterious effect on the protective function of HDL [9]. PON1 has been the focus of intensive research and has been reported as an independent risk factor for CVD [10]. The most convincing data to link PON1 with heart disease comes from transgenic mouse studies. HDL from PON1 ‘knockout’ mice have an increased capacity to oxidize LDL and are more prone to develop atherosclerosis than their wild-type siblings as a consequence of increased oxidative stress [11].

Two single nucleotide polymorphisms in the PON1 coding region affect the anti-atherogenic properties and have been associated with risk for CVD [12], a substitution of glutamine (Q) for arginine (R) at position 192 (Q192R) and a substitution of leucine (L) for methionine (M) at position 55 (L55M) [13-16]. The Q192R polymorphism alters the enzyme’s ability to protect LDL from oxidation in vitro with 192Q is the most protective, while L55M polymorphism affects the enzyme concentration with 55 M is associated with lower serum concentration [17].

Data from several studies showed ethnic variations in the interpretation of CVD associated with PON1 polymorphisms [18-21]. The aim of this work is to investigate PON1 Q192R and L55M polymorphisms in Egyptian patients with type 2 diabetes mellitus (T2DM) and its association with CVD.

## Materials and methods

### Subjects

The studied subjects were recruited from the Outpatients’ Clinic of the National Research Center and National Diabetes & Endocrinology Institute, Cairo, Egypt. Data of family and medical history, smoking habits and physical activity was obtained by questionnaire. Cigarette smokers were considered if they had once smoked even if they were no longer smokers, and physical activity was defined as exercise for 2–3 days/week for at least 30 minutes. Clinical examination including systolic blood pressure (SBP) and diastolic blood pressure (DBP) measurements was applied. Anthropometric measurements (weight and height) were collected and used for BMI calculation according to the standard formula BMI = weight (kg)/[height (m)]^2^. Hypertension was defined as blood pressure above 140/90 mmHg or taking antihypertensive drugs. According to the criteria of American Diabetes Association Classification 2010 [22], subjects were classified into 3 groups:

#### Normal healthy control group

Included 50 subjects with fasting plasma glucose (FPG) < 100 mg/dL. Exclusion criteria were hyperlipidemia, hypertension, family history of any form of CVD, diabetes mellitus, hepatic and renal diseases, endocrine disease, metabolic disorders, autoimmune diseases and those under medication.

#### T2DM patients without CVD

Included 68 subjects fulfilled the diabetes mellitus diagnostic criteria of FPG ≥ 126 mg/dL or were under antidiabetic treatment (oral and/or insulin) with no history or signs of any CVD. Exclusion criteria included renal disease, hepatic disease, endocrine disease, other metabolic disorders and autoimmune diseases.

#### T2DM complicated with CVD

Included 66 subjects diagnosed to have diabetes with FPG ≥ 126 mg/dL or under antidiabetic treatment and complicated with any of the vascular disease e.g. ischemic heart disease (IHD), macroangiopathy and/or cerebrovascular disease. Exclusion criteria included renal disease, hepatic disease, endocrine disease, other metabolic disorders and autoimmune diseases.

Informed consent was obtained from all subjects and the study protocol was approved by the Ethics Committee of the National Research Center.

## Methods

### Lipid analysis and biochemical markers

Venous blood samples were collected from all subjects after 12 hours of overnight fast. Total cholesterol (TC), Triglycerides (TG), high density lipoprotein (HDL-C), glucose and glycosylated hemoglobin (HbA1c) were quantified using an automated clinical chemistry analyzer Roche Diagnostics (Germany). Low density lipoprotein (LDL-C) level was calculated using Friedewald formula [23].

### Genotyping of PON1 Q192R and L55M polymorphisms

Genomic DNA was isolated from whole peripheral blood using QIAamp DNA extraction kit (Qiagen Hilden, Germany, Cat no. 51304) according to the manufacturer’s protocol.

L55M (rs854560) and Q192R (rs662) polymorphisms of the PON1 gene were determined by Taqman-based allelic discrimination assay using the following primers and probes: For L55M: 5′-TTTCTGTTCTCTTTTCTGGCAGAAA-3′ forward and 5′-GAAAACACTCACAGAGCTAATGAAAGC-3′ reverse; 55 M: 5′-FAM-CCATTAGGCAGTATCTCCATGTCTTCAGAGCC-3′ and 55 L: 5′- VIC-TCCATTAGGCAGTATCTCCAAGTCTTCAGAGCC-3′. For Q192R: 5′-GGACCTGAGCACTTTTATGGCA-3′ forward and 5′-GACAACATACGACCACGCTAAACC-3′ reverse; 192Q: 5′-FAM-TTCTTGACCCCTACTTACAATCCTGGGAGATGT-3′ and 192R: 5′-VIC-CTTGACCCCTACTTACGATCCTGGGAGATGT-3′.

All primers and probes were designed by Applied Biosystems. Genotyping assays were performed on ABI 7500 Real Time PCR (Applied Biosystems). The genotyping success rates were greater than 95% for all SNPs. For the genotyping quality control, 10% of samples were randomly selected and measured in duplicates and the concordance rate was 100%.

### Assay of PON1 enzyme concentration in serum

Serum PON1 concentration was measured by enzyme linked immunosorbent assay (ELISA) using Human Paraoxonase-1 (PON1) ELISA kit Cat# 95462 (Glory Science Co., Ltd, TX, USA).

### Statistical analysis

Data were analyzed using SPSS version 16.0 for Windows (Chicago, IL, USA). Chi-square was used to test the difference in genotypes frequency between the groups. Means of serum PON1 concentration according to different genotypes were compared using analysis of variance (ANOVA) and t-tests. Logistic regression analysis and odds ratios were calculated to test the association of genotypes and CVD, keeping wild type as the reference. The association of Q192R polymorphism with other CVD risk factors was tested by multiple logistic regression analysis, assessing independence from potential confounders. Variables such as age, male gender, smoking, BMI, SBP, TC, HDL, LDL and TG were included into the logistic model. P value less than 0.05 was considered significant.

## Results

Our study included 184 subjects classified into 68 patients with T2DM, 66 patients with T2DM+CVD, and 50 control subjects. Our CVD patients were as follows: 45 (68%) had ischemic heart disease (IHD), 7 (11%) had cerebrovascular disease, 8 (12%) had macroangiopathy, 4 (6%) had combined IHD and cerebrovascular, and 2 (3%) had combined macroangiopathy and cerebrovascular disease.

### The general characteristics and biochemical variables of the study population

Demographic, clinical and biochemical data of different groups is shown in Table 1. A significant age difference was found between controls and CVD group (p < 0.01). Also a significant sex difference was found between controls and CVD group (p = 0.01) due to the high prevalence of males among our CVD patients. Diabetic patients had lower levels of physical activity (p = 0.01) as compared to controls and patients with CVD had lower levels of physical activity when compared to patients without CVD (p = 0.005). There was a significant association between CVD and the duration of diabetes (p = 0.004). Other clinical data such as BMI, SBP and DBP were significantly higher in both groups when compared to controls. CVD patients had higher frequency of hypertension (p = 0.01) as compared with patients without CVD.

**Table 1:** **General characteristics and biochemical variables of the study population**

**Variable**	**Controls (n=50)**	**T2DM (n=68)**	**T2DM+CVD (n=66)**
**Age (Years)**	51.24 ± 7.62	51.75 ± 6.00	58.20 ± 7.12**
**Sex (male/female)**	27/23	38/30	50/16**
**BMI (kg/m2)**	23.21 ± 4.62	27.59 ± 4.90*	29.81 ± 5.55**
**SBP (mmHg)**	121.90 ± 15.29	131.67 ± 20.60*	144.14 ± 21.16**^†^
**DBP (mmHg)**	81.19 ± 8.93	88.75 ± 16.05*	90.14 ± 9.27**
**Hypertension (%)**	-	33.3	94.3^†^
**Smokers (%)**	9.5	11.1	14.3
**Physical activity (%)**	57.1	58.3	25.7**^†^
**Diabetes duration (Years)**	-	8.50 ± 7.41	13.00 ± 6.97^†^
**Glucose (mg/dL)**	84.90 ± 9.61	146.83 ± 58.31*	164.00 ± 68.27**
**HbA1c (%)**	5.42 ± 0.61	6.30 ± 1.19*	6.61 ± 1.37**
**Triglyceride (mg/dL)**	117.57 ± 31.28	143.61 ± 73.14*	169.00 ± 66.43**^†^
**TC (mg/dL)**	186.86 ± 28.68	193.92 ± 50.59*	206.71 ± 44.98**^†^
**LDL-C (mg/dL)**	111.95 ± 15.95	129.22 ± 42.05*	141.60 ± 45.04**^†^
**HDL-C (mg/dL)**	55.71 ± 11.24	49.93 ± 11.26*	41.97 ± 12.79**^†^
**Serum PON1 (nmol/L)**	18.87 ± 17.04	7.82 ± 5.44*	9.22 ± 14.94**

Significance was confirmed by Bonferroni test.

*Significant p in comparison between controls and T2DM, **Significant p in comparison between controls and T2DM+CVD, ^†^Significant p in comparison between T2DM and T2DM+CVD.

BMI: body mass index; SBP: systolic blood pressure; DBP: diastolic blood pressure; HbA1c: hemoglobin A1c; TC: total cholesterol; LDL-C: low density lipoprotein cholesterol; HDL-C: high density lipoprotein cholesterol; VLDL-C: very low density lipoprotein cholesterol; PON1: paraoxonase-1.

The mean of serum PON1 enzyme concentration was significantly lower in patient groups compared to controls (p = 0.006). No significant difference was found between diabetic patients with CVD and those without CVD (p>0.05).

### PON1 polymorphisms genotype and allele distribution in patients vs. controls

Analysis of genotypes and alleles distribution of Q192R and L55M polymorphisms in T2DM, with and without CVD, and control subjects is shown in Table 2. Data revealed that the genotype and allele frequencies of Q192R were significantly different between controls and diabetic patients. Genotype 192QQ was detected in 66% of controls vs. 44.8% of diabetic patients, while QR and RR genotypes were more frequent in diabetic patients than in controls (44% and 11.2% vs. 24% and 10%, respectively), p = 0.02. Allele Q was found in 78% of controls vs. 66.8% of patients, while allele R was more frequent in patients (33.2%) than in controls (22%); p = 0.03.

**Table 2:** **Genotypes and alleles frequency of PON1 Q192R and L55M polymorphisms in patients and control groups**

	**Control N=50**	**Patients N=134**	**OR (95% CI)**	**P value**
**Q192R**				
QQ	33 (66%)	60 (44.8%)	Reference	0.02
QR	12 (24%)	59 (44%)	2.1 (1.2–4.8)	
RR	5 (10%)	15 (11.2%)	3.2 (1.3–6.6)	
**L55M**				
LL	7 (14%)	23 (17.2%)	Reference	0.009
LM	14 (28%)	66 (49.3%)	2.1 (1.4–5.5)	
MM	29 (58%)	45 (33.5%)	2.8 (1.7-6.2)	
**Allele**	**2n=100**	**2n=268**		
192Q	78 (78%)	179 (66.8%)	Reference	0.03
192R	22 (22%)	89 (33.2%)	1.9 (1.2–3.2)	
55 L	28 (28%)	112 (42%)	Reference	0.01
55 M	72 (72%)	156 (58%)	2.0 (1.1–3.5)	

As regards L55M, the frequency of genotype MM was higher in controls (58%) than in patients (33.5%), while the frequency of genotypes LM and LL were higher in patients than in controls (49.3% and 17.2% vs. 28% and 14%, respectively); p = 0.009. Allele M was found in 72% of controls vs. 58% of patients, while Allele L was more frequent in patients than in controls (42% vs. 28%); p = 0.015.

### PON1 polymorphisms genotype and allele distribution in T2DM patients with and without CVD

Analysis of genotypes and alleles distribution of Q192R and L55M polymorphisms in T2DM and T2DM + CVD patients is shown in Table 3. Results showed a significant difference between the two groups with QQ genotype more frequent in T2DM patients than in T2DM + CVD patients (57.4% vs. 32%), while the frequency of QR and RR genotypes were significantly increased in T2DM + CVD (54.4% and 13.6% vs. 33.8% and 8.8%, respectively), p = 0.01. Allele Q was detected in 74% of patients without CVD vs. 59% of patients with CVD, while allele R was more frequent in patients with CVD (41%) than in patients without CVD (26%); p = 0.008. No statistical significant difference was found between the two groups as regards L55M genotype distribution (p = 0.9) or alleles frequency (p = 0.8).

**Table 3:** **Genotypes and alleles frequency of PON1 Q192R and L55M polymorphisms in T2DM and T2DM+CVD patient groups**

	**T2DM N = 68**	**T2DM+CVD N=66**	**OR (95% CI)**	**P value**
**Q192R**				
QQ	39 (57.4%)	21 (32%)	Reference	0.01
QR	23 (33.8%)	36 (54.4%)	1.7 (1.0–3.8)	
RR	6 (8.8%)	9 (13.6%)	2.1 (1.3–4.6)	
**L55M**				
LL	11 (35%)	12 (41%)	Reference	0.9
LM	34 (47%)	32 (45 %)	1.08 (0.8–1.4)	
MM	23 (18%)	22 (14%)	0.99 (0.7–1.2)	
**Allele**	**2n=136**	**2n=132**		
192Q	101 (74%)	78 (59%)	Reference	0.008
192R	35 (26%)	54 (41%)	1.3 (1.0–2.7)	
55 L	56 (59%)	56 (64%)	Reference	0.8
55 M	80 (41%)	76 (36%)	0.9 (0.8–1.1)	

### Effects of Q192R and L55M polymorphisms on PON1 serum enzyme concentration

Means of serum PON1 concentration according to different genotypes are shown in Table 4. Q192R polymorphism associated with low serum PON1 concentrations, and lowest concentrations were observed in QR and RR genotypes (p = 0.04). L55M did not associate with significant difference in enzyme concentration. Though patients with 55 L allele had higher enzyme concentrations, yet that was of no statistical significance (p = 0.18).

**Table 4:** **Means of serum PON1 concentration in different genotypes of Q192R and L55M polymorphisms**

	**Serum PON-1 mean±SD (nmol/L)**	**P value**
192 QQ	12.97 ± 15.75	0.04
192QR+192RR	8.95 ± 10.2	
55MM	9.07 ± 6.22	0.18
55LM+LL	11.80 ± 15.77	

### Association of Q192R with CVD in T2DM patients

Multiple logistic regression analysis was used to investigate potential correlations of 192R allele with other cardiovascular risk factors: age, male gender, smoking, BMI, SBP, TC, HDL, LDL and TG. We demonstrated statistical significant correlations between 192R and age (ORs 1.39, 95% CI 1.1–1.73, p = 0.04), male gender (ORs 0.94, 95% CI 0.45–0.86, p = 0.016), SBP (ORs 1.92, 95% CI 1.38–2.78, p = 0.007), TC (ORs 0.97, 95% CI 0.36–0.86, p = 0.016), HDL (ORs 2.21, 95% CI 1.38–2.79, p = 0.001) and LDL (ORs 3.20, 95% CI 2.31–6.58, p = 0.001) and TG (ORs 0.54, 95% CI 0.36–0.86, p = 0.012). No significant correlations were detected with smoking (ORs 0.34, 95% CI 0.29–0.79, p = 0.23) or BMI (ORs 0.54, 95% CI 0.24–0.63, p = 0.12).

## Discussion

Oxidative modification of LDL is believed to be a major triggering event in the initiation and progression of atherosclerosis and cardiovascular events [24]. Recently, PON1 became the focus of intense research after the identification of its antioxidant properties, particularly its capacity to protect LDL from oxidative damage.

In the present study, we investigated the association of PON1 Q192R and L55M polymorphisms with the CVD in T2DM in Egyptian patients. We found an association of 192R and 55 L alleles with T2DM. Serum PON1 enzyme concentration was significantly reduced in our diabetic patients compared to controls and lowest enzyme concentrations were associated with the 192R allele. As regards the CVD, a significant association with 192R allele was demonstrated. Serum concentration of PON1 was not significantly different between diabetic patients with CVD and those without CVD. Allele 55 L was previously reported to be associated with higher concentrations of the enzyme [25], but lower enzyme activity [18]. L55 isoform was found to be more stable and resistant to proteolysis [26]. This could explain the relatively higher serum enzyme levels found in our L allele carrier patients.

Our findings support the previously reported hypothesis that reduced PON1 concentration and/or activity might play a role in the pathogenesis of T2DM itself. It is suggested that oxidative stress induced by reduced PON1 concentration and/or activity results in reducing glucose uptake from blood by muscle cells and develops into insulin resistance. Results from previous studies in which decreased PON1 activity and concentration has been linked with impaired glucose tolerance [27,28] and increased insulin resistance in healthy subjects [29,30] also support this hypothesis. Reduced PON1 concentration and activity in Type I and Type II diabetic patients has been reported in many studies [31-36].

Logistic regression analysis with other independent risk factors revealed an independent correlation of 192R with CVD risk. The Q192R polymorphism in the PON1 gene hampers the ability of PON1 to inhibit LDL oxidation, suggesting that the 192R allele is less effective than 192Q in preventing LDL oxidation [37,38]. Thus, carriers of the R allele are more susceptible to develop CVD than carriers of the Q allele. Randa et al. [39] reported that individuals with PON1 RR genotype have 9-fold risks to develop CAD in Egyptians while those with the PON1 QR genotype have 4-fold risks. Also, the risk factors for CAD (diabetes, hypertension, dyslipidemia) were significantly associated with PON1 RR genotype.

Lower PON1 activity was associated with carotid arteriosclerosis and cerebral atherosclerosis in stroke patients [13], and was reported as a risk factor for CVD in systemic lupus patients [14] and CAD in type 2 diabetes in North-West Indians [15]. Also, an association between PON1 polymorphisms and CAD risk was demonstrated in Thai population, where the frequencies of 192R allele and 192RR genotype, as well as, 55 M allele and 55LM genotype were significantly higher in CAD patients [16].

In a study by Ito et al. [40] involving the PON1-192R allele and the environmental risk factors, PON1-192R allele was reported as the most predictive independent risk factor for coronary spasm followed by cigarette smoking. Also, plasma levels of TBARS (Thiobarbituric acid-reactive substances), markers of oxidative stress, were higher in RR than in QQ genotypes suggesting that PON1-192R allele may play an important role in the genesis of coronary spasm, probably by attenuating the suppression of oxidative stress.

In a recent study; including PON1 genotyping, activity and lipid profile and their association with significant coronary stenosis (SCS) in Tunisian population, PON1 activity was lower in patients with SCS than in controls. Low activity was associated with 192R and 55 L alleles. In the presence of diabetes, PON1-192RR genotype associated with an increased risk of SCS while 55MM genotype associated with lower risk [18]. In contrast, in a study on 589 patients (419 Caucasian, 120 South Asian, 50 other) to determine factors which modulate serum PON1 in type 2 diabetes, PON1 activity associated negatively with insulin resistance, triglycerides and 55 M allele; and positively with 192R allele. While PON1 concentration associated negatively with Caucasian ethnicity and duration of diabetes, and positively with 192R allele [19].

Altuner et al. declared that RR genotype associated with higher PON activity than QQ or QR genotypes, while LL genotype associated with higher PON activity than MM genotype in Turkish population [20]. In Asian Indians, CAD was associated PON1 192R allele. Haplotype LQ (55 L and 192Q) showed protective effect, while haplotype MR (55 M and 192R) was associated with increased risk of CAD [21]. A recent meta-analysis of 88 studies comprising 24,702 CAD cases and 38, 232 controls revealed a significant association of Q192R polymorphism with the disease risk [41]. The controversial data in the interpretation of CVD association with PON1 polymorphism might be attributed to ethnic variation, sample size, patients’ recruiting criteria, gene–gene and gene–environment interactions [42-45].

## Conclusion

Our results indicate that PON1 192R and 55 L alleles are associated with T2DM, and suggest that they may play a role in the pathogenesis of the disease. Q192R is associated with CVD and lower serum enzyme concentration and might represent a novel risk factor for CVD in Egyptian patients with T2DM.
